# Addressing pediatric traumatic brain injury in Brazil: a call for targeted prevention

**DOI:** 10.1055/s-0045-1806814

**Published:** 2025-04-22

**Authors:** Gisele Sampaio Silva

**Affiliations:** 1 Universidade Federal de São Paulo, São Paulo SP, Brazil.; 2 Hospital Israelita Albert Einstein, São Paulo SP, Brazil.

Traumatic brain injury (TBI) remains a significant public health concern worldwide, particularly among pediatric populations. In Brazil, ∼20% of TBI victims are children and adolescents, with nearly 1,000 deaths reported annually. 1 The recent study conducted in São Paulo underscores the pressing need for enhanced preventive strategies, especially in socioeconomically vulnerable metropolitan areas. 2

## The epidemiology of pediatric TBI: a Brazilian perspective

The present study showed that children and adolescents hospitalized for TBI in São Paulo predominantly come from the northern metropolitan region, an area marked by significant socioeconomic disparities. 2 Domestic accidents, including falls from heights, were the leading cause of TBI among children under nine years old, while older children were more frequently victims of pedestrian traffic accidents. These findings align with international trends, according to which younger children face increased risks from household hazards, and school-aged children experience a shift in risk exposure toward outdoor environments. 3 4

Moreover, the study highlights a gender disparity, with boys accounting for 71% of cases. 2 This pattern is consistent across multiple studies and is often attributed to behavioral and societal factors that result in higher-risk activities among male children. 1 3 5 Addressing this disparity requires a multi-faceted approach that includes education, supervision, and modifications in high-risk environments.

One of the most critical insights from the study is the relationship between TBI incidence and socioeconomic conditions. The northern and eastern zones of São Paulo, where most cases originate, have some of the city's lowest human development index (HDI) scores. 2 Lower-income families often reside in high-density housing with inadequate safety measures, increasing the risk of falls and other injuries. Furthermore, children in these communities may be more exposed to traffic hazards due to insufficient pedestrian infrastructure. 6

International research corroborates that socioeconomic factors significantly impact TBI outcomes. Children from low-income backgrounds often experience delays in receiving medical care, have limited access to rehabilitation services and face a higher likelihood of long-term cognitive and functional impairments. 7 This reinforces the urgent need for public policies that address health inequities by implementing region-specific prevention programs.

Preventive interventions must be tailored to the specific risks associated with different age groups and socioeconomic contexts to mitigate the burden of pediatric TBI. Countries with successful TBI prevention programs have adopted a combination of public education, legislation, and environmental modifications. 8 Brazil can benefit from these international models while adapting them to local needs.

- Home safety initiatives: interventions to reduce falls among young children should prioritize low-cost home modifications, such as window guards, safety gates, and improved supervision practices. Educational programs targeting caregivers can enhance awareness of common household hazards. 9
- Traffic safety programs: given the high incidence of traffic-related TBIs in older children, improved pedestrian infrastructure, speed regulation in school zones, and stricter enforcement of traffic laws are necessary (10). Helmet laws for bicyclists and motorcyclists should be expanded and rigorously enforced. 6 8
- Community-based education: outreach initiatives tailored to high-risk neighborhoods can empower communities with knowledge about TBI prevention. Schools, local health centers, and community leaders can play a pivotal role in disseminating safety information and advocating for safer environments. 10
- Legislative actions: strengthening building codes to require childproofing measures in residential buildings, particularly in low-income areas, can significantly reduce the occurrence of falls from heights. Additionally, urban planning policies should integrate safety considerations into the design of public spaces. 8

Medical professionals, particularly those working in trauma centers, play a vital role in both the immediate care and long-term rehabilitation of TBI patients. Early identification of at-risk populations allows for targeted interventions to prevent recurrent injuries. Moreover, researchers should continue to investigate the epidemiological trends of TBI, evaluating the effectiveness of implemented policies and adjusting strategies accordingly. 8 Collaboration between healthcare providers, policymakers, and community organizations is essential to transforming research findings into actionable solutions. By prioritizing pediatric TBI prevention, Brazil can reduce the burden on its healthcare system, improve the quality of life for affected children, and ultimately save lives.

In conclusion, the study from Tude Melo et al. in São Paulo provides a critical lens into the epidemiology of pediatric TBI in Brazil, reinforcing the need for region-specific, socioeconomic-sensitive prevention strategies. Brazil can significantly reduce the incidence of pediatric TBI by adopting a multi-pronged approach that includes home safety measures, traffic regulations, community education, and legislative action. As stakeholders in public health, we must advocate for policies that address the root causes of these injuries, ensuring a safer future for all children.
